# Is the insula linked to sleep? A systematic review and narrative synthesis

**DOI:** 10.1016/j.heliyon.2022.e11406

**Published:** 2022-11-05

**Authors:** Yangyang Wang, Manli Li, Wenchao Li, Lifei Xiao, Xianhao Huo, Jiangwei Ding, Tao Sun

**Affiliations:** a Ningxia Key Laboratory of Cerebrocranial Disease, Ningxia Medical University, Yinchuan, Ningxia, China; b Sanquan College of Xinxiang Medical University, Xinxiang, Henan, China; c Department of Neurosurgery, The First Affiliated Hospital of Zhengzhou University, Zhengzhou, China

**Keywords:** Insula, Sleep, Insomnia, Sleep deprivation, Sleep disorder

## Abstract

**Background:**

Sleep is critical to human beings in a surprisingly diverse set of ways, and there is, thus, continual investigation into the mechanisms of sleep. Although current studies have confirmed that multiple brain regions are involved in the regulation of both sleep and wakefulness, the association between certain important brain regions such as the insula and sleep is still unclear.

**Objective:**

The purpose of this study was to systematically review studies on the insula and sleep and to discuss the relationship between the insula and sleep.

**Methods:**

We searched the PubMed and Web of Science Core Collection (WoSCC) for articles on sleep and the insula. The time span was from inception to June 30, 2022. The search results were then narratively summarized.

**Results:**

A total of 939 studies were identified in the PubMed and WoSCC of which 115 studies were finally included in the narrative synthesis. These 115 studies can be roughly divided into 41 studies on insomnia, 39 on sleep deprivation, 33 on sleep-related experiments examining the insula, and 2 studies using basic experiments.

**Conclusion:**

The combined findings of many sleep-related studies have confirmed a close link between the insula and sleep loss, including insomnia, sleep deprivation, sleep-related disorders, and more. Although these results do not directly confirm that the insula is involved in sleep, a overall analysis of the results indicates that the insula may be a potential key brain region involved in sleep.

## Graphical abstract

**Figure:**
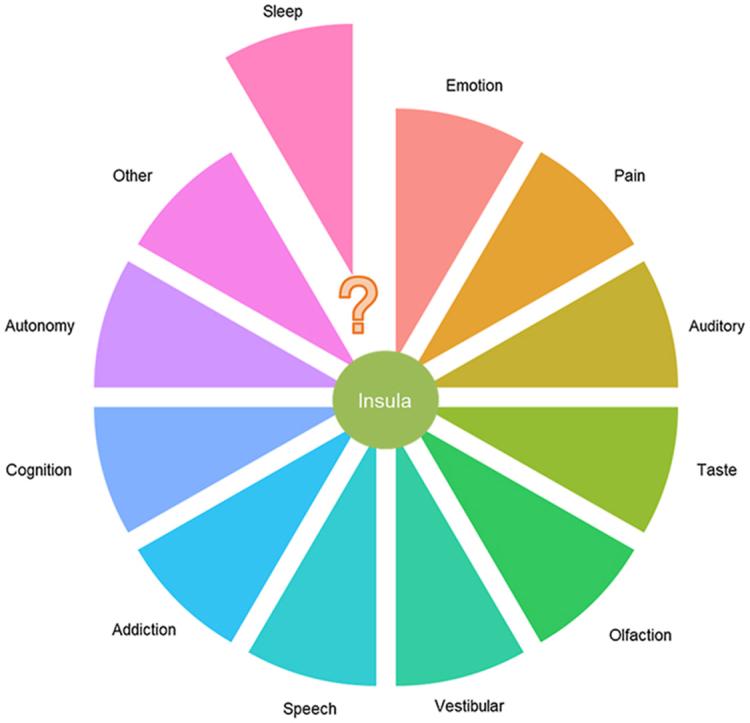

## 1 Introduction

Sleep is essential for the maintenance of normal body activities and functions and accounts for approximately one-third of our lives. Adequate sleep regulates the body systems and affects a variety of physiological functions, such as body temperature, blood pressure, heart rate, respiration, and hormone secretion [1, 2, 3, 4]. However, sleep-related health problems have gradually increased in recent years [5, 6]. Numerous studies have shown that short-term sleep loss can affect cognitive and emotional performance, and long-term sleep loss is associated with neurodegeneration [7, 8, 9, 10]. In addition, sleep-related health problems have also gradually evolved into serious social public health problems [11, 12].

Given the importance of sleep to the body, sleep mechanisms have been intensively investigated. Sleep has been shown to involve many regions and nuclei in the brain, including subcortical nuclei such as the hypothalamus, brainstem, and basal forebrain, as well as the prefrontal cortex, motor cortex, anterior cingulate cortex (ACC), and primary visual cortex [13, 14, 15, 16, 17, 18]. However, these findings have raised new questions, such as why so many nuclei as well as the cerebral cortex are involved in the maintenance of sleep or wakefulness while the detailed mechanisms underlying sleep and wakefulness are incompletely understood. Additional questions are whether, in view of the importance of sleep, the brain requires multiple switches to induce sleep, and whether other as yet undiscovered regions of the brain are involved in the control of sleep and wakefulness.

The insula has a unique anatomical location in the brain deep within the Sylvian fissure, inferior to the frontal lobe, medial to the temporal lobe, and anterior to the parietal lobe [19]. As described above, sleep has different effects on the brain, affecting both systemic and autonomic physiology, and sleep loss can lead to cognitive impairment, emotional disturbances, and hyperalgesia, among other adverse effects. The insula also participates in emotion, pain, and cognitive and autonomic functions, suggesting an overlap between the role of sleep and the function of the insula. This invites the question of whether there is an intrinsic link between the insula and sleep.

In recent years, thanks to the development of imaging, electroencephalography (EEG), and other technologies, the role of the insula in relation to sleep has received more attention. The aim of the present study was to review the published research on the association between the insula and sleep to better understand the relationship between the two and provide a reference for future studies.

## 2 Methods

### 2.1 Literature search

**Figure 1:**
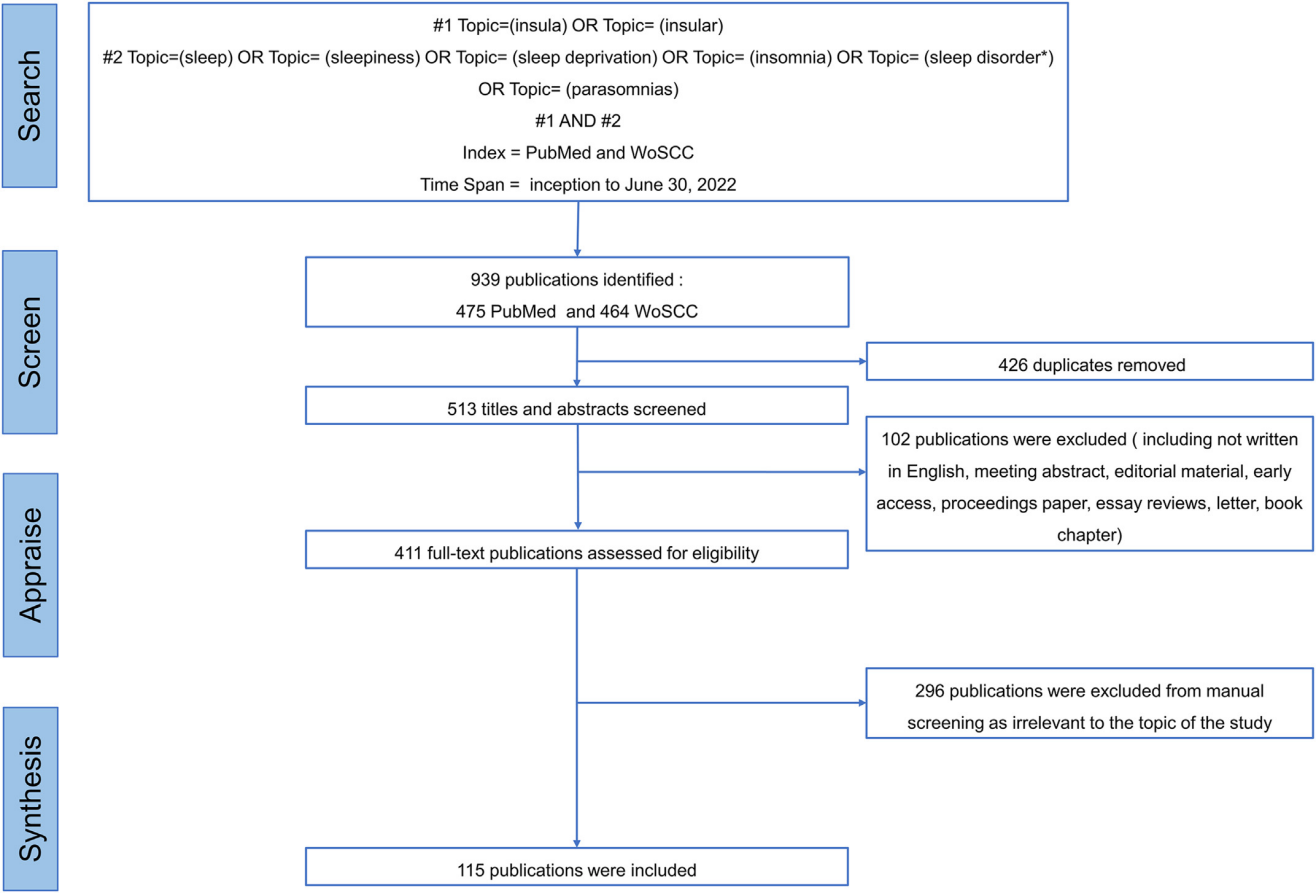
Flowchart of the search process and study selection.

Data were retrieved from the PubMed and WoSCC, an extended version of the Science Citation Index, using advanced search strategies. The following search terms were entered in the topic field: (“insula” OR “insular”) AND (“sleep” OR “sleepiness” OR "sleep deprivation" OR "insomnia" OR "sleep disorder" OR "parasomnias"). The time span was from inception to June 30, 2022 (Figure 1).

### 2.2 Inclusion and exclusion criteria

Our objective was to investigate the role of the insula in sleep. Therefore, the criteria included all articles involving the insula and sleep or sleep-related disorders. We excluded articles that were not written in English. In addition, the article type was limited to original articles, and reviews, meeting abstracts, editorial material, early access, proceedings papers, essay reviews, letters, book chapters, and experimental articles without information related to the topic were excluded. Yangang Wang and Manli Li independently screened all retrieved literature separately. Discrepancies were discussed and decided by Sun Tao or between all three investigators.

## 3 Results

### 3.1 Study selection

Of the 939 publications identified in the initial PubMed and WoSCC search, 115 studies were finally included in the narrative synthesis (Figure 1).

### 3.2 Study characteristics and findings

**Figure 2:**
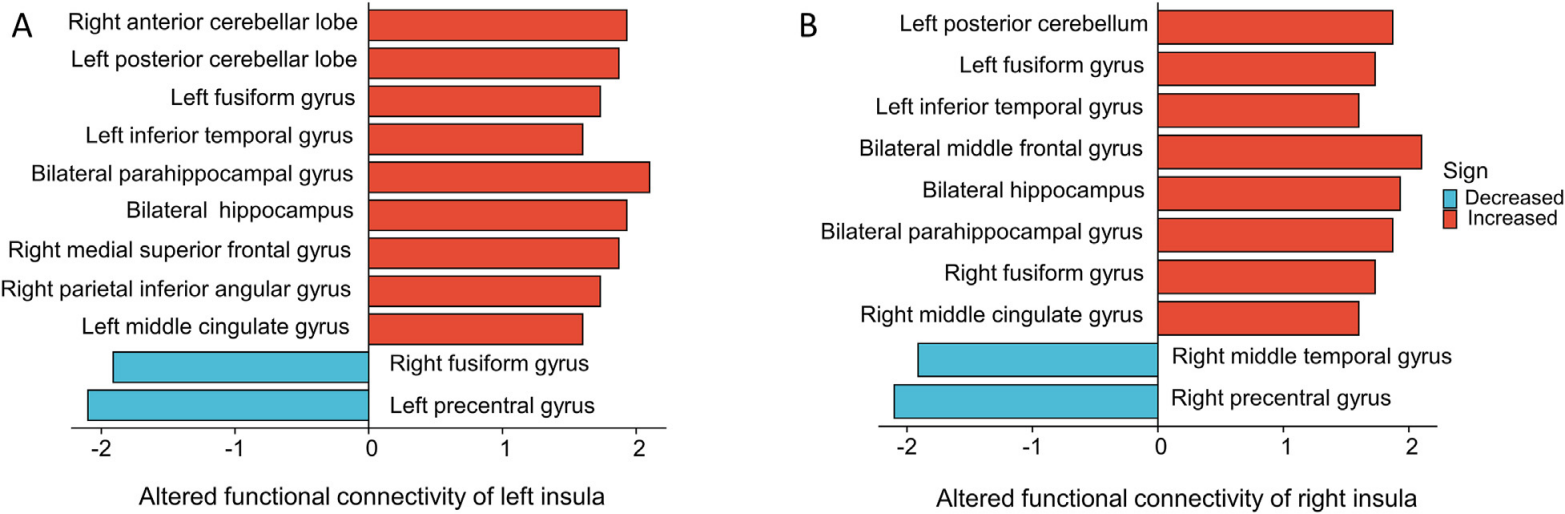
Regions reported to show altered functional connectivity with the insula in previous studies. (A) Altered functional connectivity of left insula; (B) Altered functional connectivity of right insula.

The 115 included articles could be roughly divided into the following four categories according to the research contents. There were 41 articles using patients with insomnia as subjects, 39 articles investigating sleep deprivation in volunteers, 33 articles describing sleep-related experiments investigating the insula, and 2 papers that investigated the participation of the insula in sleep using animal experiments. Most of the experiments in these 115 articles used functional magnetic resonance imaging (fMRI) technology. Therefore, we have summarized articles evaluating the role of the insula in sleep-related experiments using fMRI metrics. Table 1 summarizes the changes in spontaneous neural activity in the insula in several recent sleep-related experiments. Figure 2 summarizes the brain regions shown to have abnormal functional connectivity (FC) with the insula. In addition, we found that only four articles specifically investigated the insula and its potential role in sleep or changes in sleep-related disorders (Table 2).

**Table 1:** Changes in spontaneous neural activity in the insula shown in recent sleep-related studies.

fMRI Metrics	Study	Subjects	Changes in the insula
**ALFF**	Chen et al. [20]	Healthy female with SD 24h	Increased ALFF in left insula
	Ji et al. [21]	Children with OSA	Increased ALFF in the right insula
	Liu et al. [22]	patients with insomnia complaints in MDD	Increased ALFF in the right anterior insula
**fALFF**	Wu et al. [23]	sleep disturbance in individuals with MDD	Increased fALFF in the right anterior insula
**ReHo**	Wang et al. [24]	patients with primary insomnia	Increased ReHo in the left insula

fMRI: functional magnetic resonance imaging; ALFF: amplitude of low-frequency fluctuation (ALFF); fALFF: fractional amplitude of low-frequency fluctuations; ReHo: Regional homogeneity; SD: sleep deprivation; OSA: obstructive sleep apnea; MDD: major depressive disorder.

**Table 2:** Sleep-related studies focusing on the insula.

Study	Subjects	Methods	Results
Qi et al. [25]	Healthy volunteers (TSD 36 h)	Utilizing fMRI	The FC between the insula and other brain regions is significantly altered after TSD.
Li et al. [26]	Primary insomnia patients	Utilizing fMRI	Abnormal efficient connections exist in the right anterior insula in patients with primary insomnia.
Levichkina et al. [27]	Cat	Electrodes for electrical stimulation and recording	Insula has a dual role in sleep and wakefulness
Chen et al. [28]	Rat	Record with sleep monitor	The anterior insula is involved in regulating sleep and wakefulness.

TSD: total sleep deprivation; FC: functional connectivity; fMRI: functional magnetic resonance imaging.

## 4 Discussion and narrative synthesis

Due to the anatomical location of the insula, little research has been done on its relationship with sleep. However, thanks to the rapid development of fMRI and EEG in recent years, we can now use neuroimaging to understand the neural mechanisms related to sleep as well as pathological changes in the relevant brain regions. In the following sections, we discuss whether the insula is involved in sleep from the aspects of insomnia, sleep deprivation, sleep-related research, and animal experiments.

### 4.1 Insomnia and the insula

Insomnia is a recognized health problem in modern society that can lead to physiological dysfunction, emotional disorders such as anxiety and depression, cognitive impairment, and increased risk of cardiovascular, nervous system, and multisystem diseases [29, 30]. Interestingly, the range of health problems caused by insomnia closely resembles the documented functions of the insula, suggesting the possibility of a connection between the two.

Regional homogeneity (ReHo), amplitude of low-frequency fluctuation (ALFF), and fractional amplitude of low-frequency fluctuations (fALFF) are commonly used metrics for analyzing fMRI data and represent spontaneous neural activity. Interestingly, abnormalities in the insula have been observed. Wang et al. observed patients with primary insomnia by rs-fMRI and found that the ReHo value of the left insula was increased and that this increased ReHo value was positively correlated with the patients’ self-reported anxiety scores [24]. In a study of patients with major depressive disorder who complained of insomnia, Liu et al. found that the ALFF was increased in the right anterior insula and that the increased ALFF value in the insula was correlated with sleep disturbance scores [22]. Chen et al. used fMRI/EEG to record the Blood Oxygen Level-Dependent (BOLD) signal and EEG gamma frequency power in 17 female insomniacs; the results suggested that the insula, as a key node of a salient network, was involved in hyperarousal in patients with insomnia [31]. These results suggest another thought. Since the subjects of these studies were all insomnia patients, it might be possible that the change in spontaneous neural activity in the insula itself leads to insomnia, or vice versa, or the related complications of insomnia such as anxiety, in turn leading to alterations in the spontaneous neural activity of the insula, which regulates emotion.

FC is an fMRI indicator that can study the interactions between different brain regions and is also often used to study the underlying neural circuitry associated with insomnia. Several recent studies of patients with insomnia have found abnormal FC between the insula and other brain regions [32, 33, 34, 35]. Unfortunately, these studies did not use the insula as the region of interest. To investigate neural mechanisms underlying insomnia, Li et al. selected the right anterior insula as the region of interest to analyze the directional FC of the insula in patients with primary insomnia (PI), revealing abnormalities in the directional FC between the right anterior insula and multiple brain regions, and that effective connectivity between the right anterior insula and the left posterior central gyrus was associated with insomnia severity [26]. In another rs-fMRI study of patients with PI, Li et al. observed that abnormal voxel-wise functional connectivity strength (FCS) in the right anterior insular cortex and left middle frontal gyrus are potential neural markers of PI [36]. These results all indicate that there are FC abnormalities between the insula and other brain regions in PI patients, suggesting that sleep loss in PI patients leads to abnormal FC in the insula and other brain regions, or vice versa.

### 4.2 Sleep deprivation and the insula

A commonly used method in sleep research for studying physiological mechanisms is to construct models for varying durations of sleep deprivation. Similar to insomnia research, sleep deprivation research commonly uses rs-fMRI. Dai et al. performed rs-fMRI scans of volunteers deprived of sleep for varying times and observed widespread changes in the gray matter volume (GMV) of the insula after acute sleep deprivation [37]. Motomura et al. found that the FC between the left insular cortex and the ACC was positively associated with subjective sleepiness [38]. Recently, Qi et al. chose the insula as the region of interest and assessed abnormal FC in the bilateral insula after sleep deprivation, proposing that the presence of abnormal insula-related circuits may be a neural biomarker of attention impairment after complete sleep deprivation [25]. Sleep deprivation can lead not only to morphological changes in the insula but also to changes in its FC with other brain regions. This is further evidence that indicates that the insula may be closely related to sleep.

There are other techniques for studying sleep deprivation and studies using different techniques have also observed abnormal changes in the insula. Using recorded EEG data, Zhang et al. showed that the effective connectivity between the right insula and the left ACC decreased after sleep deprivation, with progressive deterioration after prolonged sleep deprivation [39]. Using positron emission tomography (PET) scans to record wakefulness during different sleep stages, Noirhomme et al. found some degree of change in the cerebral blood flow (CBF) in the insular cortex between wakefulness and non-rapid eye movement (REM) sleep states [40].

### 4.3 Sleep-related experiments examining the insula

Various sleep studies have reported changes in the insula. In a study of 37 firefighters, Park et al. found that reduced cerebral blood flow in the insular cortex was associated with poor sleep quality [41]. Yin et al. found that changes in the GMV in the right insula were associated with sleep quality in college-age volunteers [42]. A similar study of sleep quality in young volunteers by Guadagni et al. revealed that BOLD signals in the left insula were increased in individuals who subjectively reported better sleep quality [43]. Although these studies have confirmed that the imaging changes observed in the insula are related to sleep quality, several studies have questioned whether the insula is involved in the physiological process of sleep. Falgàs et al. found that changes in the insular GMV were related to the severity of sleep loss in a comparison of insomnia and non-insomnia groups; however, no differences were seen when the volumes were compared [44]. Coincidentally, van den Heuvel et al. found that maternal stress during pregnancy can alter fetal insula-cerebellum connectivity and increase sleep problems in children after birth; however, further analysis did not find that this change in FC was associated with sleep problems in children [45]. Similarly, Kim et al. found that bilateral activation of the anterior insula occurred during sleep disturbance caused by negative life stress, although the degree of activation of the anterior insula did not correlate with the severity of sleep disturbance [46]. Although the results of these studies appear to be contradictory, they all confirm that imaging changes in the insula coincide with sleep problems. The inconsistent results of these studies may be due to differences in the subjects used in the studies, suggesting that the insula may play different roles in specific sleep problems in different populations.

Sleep disturbances have also been observed in cases where disease or pathological changes have affected the insula. Proserpio et al. used stereo EEG in patients with sleep-related hypermotor epilepsy to show that their seizures may have originated in the insula [47]. Byun et al. suggested that altered FC of the insula may be an underlying neural mechanism of isolated rapid eye movement sleep behavior disorder (IRBD) [48]. Koenigs et al. reported that patients with focal brain lesions involving the frontal insula had a higher incidence of insomnia [49] while Branger et al. suggested that atrophy of the left insula may underlie difficulties in initiating or maintaining sleep [50]. Research has also shown that the insula is involved in obstructive sleep apnea (OSA) and abnormal FC of the insula with various subregions has been reported in OSA patients [51]. These findings suggest that when neurological diseases are associated with insomnia, we should focus our attention on the insula, suggesting that it may be a potential target for the treatment of insomnia. For instance, Shi et al. have suggested that low-frequency repetitive transcranial magnetic stimulation can improve the symptoms of insomniac patients, related to weakened baseline connectivity of the beta and alpha-band networks between other cortical regions such as the right dorsolateral prefrontal cortex and insula [52].

### 4.4 Research on insula participation in sleep in basic experiments

Basic experiments investigating the involvement of the insula in sleep are rare. Chen et al. reported that rats with lesions of the anterior insula showed increased REM sleep and non-REM sleep time, together with a reduced duration of sustained wakefulness [28]. Levihkina et al. reported efficient afferent and efferent connectivity patterns in the insular cortex during the transition from wakefulness to sleep by studying neuronal responses in the cat insular cortex following electrical stimulation of the gut wall during wakefulness and natural sleep. The observed changes demonstrated the dual function of the insula in sleep and wakefulness [27]. From these two basic experiments, it can be concluded that the role of the insula in sleep is complex, which may be because the insula is considered an important information transfer station in the brain.

### 4.5 Open questions and outlook

The studies described above point to a close relationship between the insula and sleep. Nevertheless, these findings do not directly demonstrate a relationship between the insula and sleep. At the same time, these findings invite the consideration of several key open questions. First, it is clearly recognized that lack of sleep can lead to impairment of both emotion and cognition, and the insula is known to be a key brain area for emotion and cognition. However, the subjects used in these studies were not in a state of normal sleep and wakefulness but a state of sleep loss. This raises the question of whether the observed changes in the imaging data and other aspects of the insula were caused by complications arising from sleep loss or whether the changes in the insula were caused by sleep loss itself.

Several techniques have recently been developed to enable whole-brain multimodal studies. In human studies, direct evidence can be obtained using Stereo-Electroencephalographic (SEEG) for direct intervention of the insula. In animal model experiments, techniques such as optogenetics and chemical genetics can be used for direct intervention in the insula to understand its impact on sleep. At the same time, the circuits between the insula and key brain regions known to be involved in sleep, such as the brainstem and hypothalamus, can also be explored to study the role of the insula in sleep. These new technologies may enable a breakthrough in the question of the involvement of the insula in sleep.

## 5 Conclusion

Overall, the combined findings of many sleep-related studies have confirmed a close link between the insula and sleep loss, including insomnia, sleep deprivation, sleep-related disorders, and more. Although these results do not directly confirm that the insula is involved in sleep, they suggest that the insula may be a potential key brain region involved in sleep.

## Declarations

### Author contribution statement

All authors listed have significantly contributed to the development and the writing of this article.

### Funding statement

This work was supported by Ningxia Brain plan-the basic and clinical research on tempo-insular neural network and brain recognizing functions (2002170101).

### Data availability statement

Data included in article/supplementary material/referenced in article.

### Declaration of interests statement

The authors declare no conflict of interest.

### Additional information

No additional information is available for this paper.
